# Functional characterization of DNAAF3-AS1 in chromatin remodeling and H3K36me3 distribution

**DOI:** 10.64898/2025.12.15.694275

**Published:** 2025-12-16

**Authors:** Anna Budkina, Anatoliy Zubritskiy, Jude Aneke, Daria Marakulina, Yulia A. Medvedeva

**Affiliations:** 1 Skryabin Institute of Bioengineering, Research Centre of Biotechnology, Russian Academy of Sciences, 117312, Moscow, Russian Federation; 2 Moscow Center for Advanced Studies, 123592, Moscow, Russian Federation

## Abstract

Long non-coding RNAs (lncRNAs) represent a diversity of transcripts that can regulate gene expression and chromatin remodelling. DNAAF3-AS1 is an lncRNA with a strong genome-wide correlation between DNAAF3-AS1 expression and the histone mark H3K36me3, according to the HiMoRNA database. To validate this association, we performed DNAAF3-AS1 knockdown in human dermal fibroblasts using antisense oligonucleotides following H3K36me3 ChIP-seq. Our results demonstrate that DNAAF3-AS1 depletion leads to a significant redistribution of H3K36me3, with increased signal in intergenic regions and the first exon, and reduced enrichment across gene bodies. Additionally, differential expression analysis revealed that DNAAF3-AS1 knockdown induces promoter switching, with downregulation of gene-body promoters downstream of TSS. These findings establish DNAAF3-AS1 as a potential regulator of H3K36me3 deposition and transcriptional architecture, providing mechanistic insight into lncRNA-mediated epigenetic control.

## Introduction

A considerable number of human cells transcribe a wide variety of long noncoding RNAs (lncRNAs), comparable in number to protein-coding genes [6]. Due to their low expression, tissue specificity, and evolutionary conservation, lncRNAs are challenging to functionally annotate [4,12]. Nevertheless, other characteristics of lncRNAs are often conserved, including synteny with neighbouring genes, similarity of short sequence fragments, and secondary structure [2]. These observations suggest that lncRNAs could potentially be functional. Additionally, indirect evidence indicates that lncRNAs are tightly regulated and participate in various molecular mechanisms.

The majority of lncRNAs have been detected to interact with chromatin and are essential for the epigenetic control of specific genomic loci, as well as the organisation of chromosomes [11,22,24,30]. The identification of functional genomic targets for chromatin-interacting lncRNAs is critical. Previously, we developed HiMoRNA [21], a multi-omics resource that integrates correlated lncRNA–epigenetic changes in specific genomic locations genome-wide to computationally predict the effects of lncRNA on epigenetic modifications and gene expression. We predicted that DNAAF3-AS1 (CTD-2587H24.5) might participate in the establishment or maintenance of H3K36me3: an essential histone mark associated with actively transcribed genes that recruit transcription elongation factors, suppress aberrant transcription initiation, regulate splicing and DNA repair [14,23,32]. In this work we dig deeper into elucidating the epigenetic regulatory mechanisms of DNAAF3-AS1 via histone modifications.

DNAAF3-AS1 is a long non-coding RNA, which is expressed most abundantly in testicular tissues, pituitary, brain, adrenal gland and heart, such as its antisense gene, but there is also a significant level of its expression in human fibroblasts and skeletal muscles [8].

DNAAF3-AS1, classified as a transcribed enhancer lncRNA in ChIA-PET network analysis, may contribute to gene regulation by facilitating chromatin-associated regulatory modules [31]. DNAAF3-AS1 has also been predicted to loop between regulatory elements such as enhancers and promoters mediated by PolII and has been annotated as an enhancer by chromHMM. In work [10], DNAAF3-AS1 was classified as a repressor lncRNA acting in *cis* based on the over-representation of its targets obtained from loss-of-function expression within its topologically associated domain.

In this work, we study the influence of DNAAF3-AS1 on the predisposition to H3K36me3, validate the distribution of H3K36me3 after DNAAF3-AS1 knockdown in fibroblasts and analyse the use of differential promoters after DNAAF3-AS1 knockdown.

## Materials and Methods

### HiMoRNA data analysis

The associations for CTD-2587H24.5 (DNAAF3-AS1) and H3K36me3 were downloaded from the HiMoRNA database [1] with an absolute correlation threshold > 0.5. Genome annotation and distance to TSS were obtained using ChIPseeker [33] v1.38.0.

### ASO Transfection Protocol

Human dermal fibroblasts (HDFb d75, adult female) were purchased from the Cell Culture Collection (IDB RAS, Russia). Cells at passages 5 to 10 were cultured under the following conditions: 5% CO_2_, 37°C, DMEM/F12 (Gibco) supplemented with 1X Pen-strep (Paneco) and 10% v/v FBS (BioWest). For lncRNA knockdown, we performed HDF transfection at a confluency of 75–80% with ASO targeting the corresponding transcript in FectoMEM medium (Bioinnlabs) with GenJect40 transfection reagent (Molecta) at a final concentration of 10 pmol ASO per 1 cm^2^ of well surface. Total RNA was extracted by direct cell lysis with ExtractRNA reagent (Evrogen). 50 ng of total RNA was reverse transcribed with the MMLV RT kit and dT_20_ primer (Evrogen). qPCR was performed using 5X master mix containing SYBR (Evrogen) with normalisation to B2M level.

### Chromatin Immunoprecipitation and Sequencing (ChIP-seq)

Chromatin immunoprecipitation followed by high-throughput sequencing was performed to investigate histone mark distribution in human dermal fibroblasts (HDF) with and without lncRNA knockdown. The ChIP was conducted using the “Cross-linking Chromatin Immunoprecipitation protocol” (www.abcam.com) with some modifications. Anti-H3K36me3 (Abcam) antibodies were used for immunoprecipitating chromatin. The input controls were similarly processed without any antibody. To construct Illumina sequencing libraries for ChIP-Seq, the recovered ChIP DNA and input DNA were used. Sequencing was carried out with 2 biological replicates for control and 2 biological replicates for knockdown for different ASOs.

### ChIP-seq Data Processing and Differential Peak Analysis

Raw reads of ChIP-Seq were processed with fastp (v0.23.2) [7], and clean reads were mapped to chr1–22, chrX, and chrY sequences from the Homo sapiens reference genome (assembly GRCh38.p14) using Bowtie2 (v2.3.4.1) software [16] with local mode. Samtools (v1.8) [9] was used to sort bam alignment files by genomic coordinates, filter uniquely mapping reads and remove PCR duplicates. MACS3 v3.0.0a6 [36] was used for peak calling, comparing each H3K36me3 ChIP sample against its respective input control. Peaks were identified with the following parameters: –broad, –broad-cutoff 0.1, –nomodel, and –extsize. The phantompeakqualtools [15] was used to estimate fragment length used for the –extsize parameter. Differential binding analysis was performed using the DiffBind v3.16.0 R package (v3.10.1) [27]. Consensus peaks from all samples were merged, and read counts were computed using dba.count with summits = FALSE, fragmentSize = 200, and minOverlap = 1. MACS3 differential peaks were annotated using the GenomicFeatures v1.58.0 [19] and peaks were assigned to the nearest transcription start site (TSS ±2 kb) using TxDb.Hsapiens.UCSC.hg38.knownGene v3.20.0 [3], and mapped to gene symbols via org.Hs.eg.db v3.18.0 [5]. The metagene plots were constructed using deeptools software [26].

### Differential expression analysis

Differential expression analysis comparing DNAAF3-AS1 knockdown and control was conducted separately for the CAGE-seq promoter counts matrix obtained in FANTOM6 [25] using DESeq2 [18] for ASO_G0267577 01 and ASO_G0267577 03 (ASO 01 and ASO 03). Promoters with a false discovery rate FDR < 0.05 and |*logFC*| > 0.1 were considered differentially expressed and selected for downstream analysis.

## Results

### DNAAF3-AS1 in human and Gm15873 transcript in mice share a conservative region

**Figure 1.**
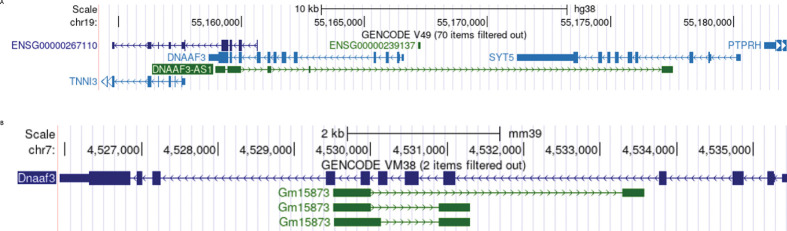
DNAAF3 (Dnaaf3) antisense genes. **A** DNAAF3-AS1 gene in human genome hg38 **B** Gm15873 in mouse genome mm39.

DNAAF3-AS1 is located on chromosome 19 and covers DNAAF3 as the antisense gene for most observed isoforms; it also covers the SYT5 gene in its longest transcript (Figure 1A). In mice, antisense for Dnaaf3 is Gm15873, located on chromosome 7 (Figure 1B). Alignment of human DNAAF3-AS1 transcripts and the Gm15873 transcript revealed a conserved region located in exon 2 of the human DNAAF3-AS1 transcript and in exon 1 of the mouse Gm15873 transcript that corresponds to the DNAAF3 exon location.

### DNAAF3-AS1 expression is linked to the redistribution of the H3K36me3 mark

**Figure 2.**
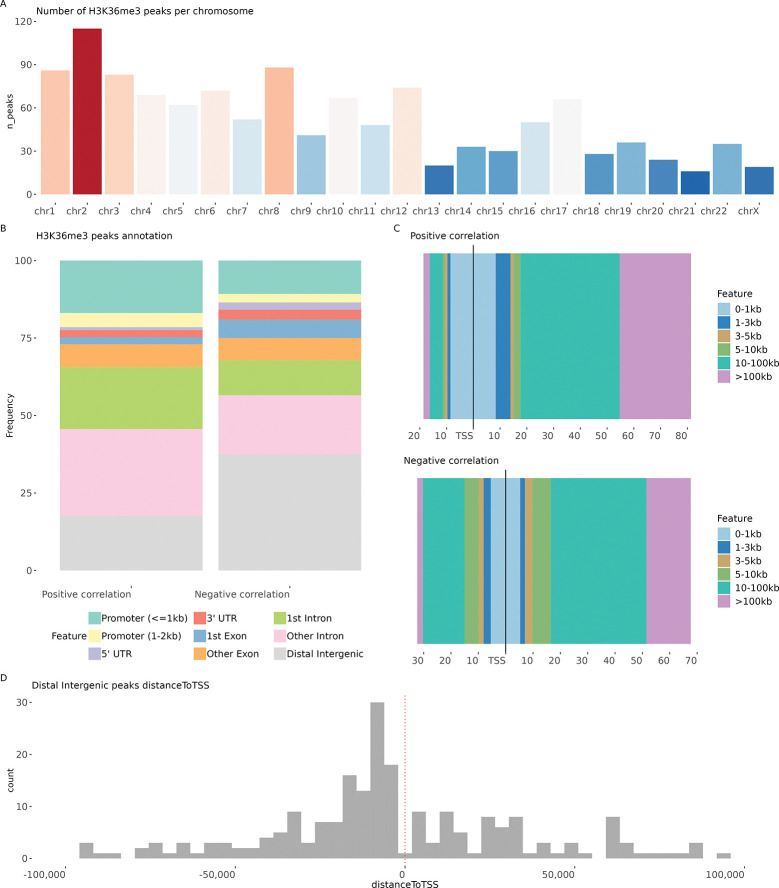
HiMoRNA results for DNAAF3-AS1 **A** Number of H3K36me3 correlated peaks per chromosome **B** Genome annotation for positively and negatively correlated peaks **C** Distribution of distance to TSS for positively and negatively correlated peaks **D** Distribution of distance to TSS for negatively correlated peaks annotated as Distal Intergenic.

The majority of DNAAF3-AS1-associated peaks (absolute Pearson correlation ¿ 0.5) in the HiMoRNA database corresponded to the histone modification H3K36me3. The distribution of the peak position (Figure 2A) shows that DNAAF3-AS1 can act as an in *trans* chromatin regulator; the highest number of peaks were located on chromosome 2.

Genome annotation of the peaks positively correlated with the expression of DNAAF3-AS1 compared to the negatively correlated peaks showed enrichment within the gene body, including a higher ratio in the introns (Figure 2B). In contrast, genome annotation of negatively correlated peaks showed that the overall ratio of such peaks was higher in distal intergenic regions and lower in introns. More negatively correlated peaks were located upstream of the transcription start site (TSS) for 5–10 kb and 10–100 kb regions compared to positively correlated peaks (Figure 2C). Most of the distal intergenic peaks were shifted upstream of TSS (Figure 2D), which may indicate activation of antisense transcription with decreased DNAAF3-AS1 expression. Additionally, a higher proportion of negatively correlated peaks were found within the first exon, potentially reflecting faster recruitment of the SETD2 complex to RNA polymerase, higher transcription rate, or premature transcription termination that limits the propagation of H3K36me3 after the first exon. The overall distribution pattern suggests that DNAAF3-AS1 expression may be linked to H3K36me3 deposition either as a regulator itself or as a target of a regulator that causes chromatin remodelling.

### DNAAF3-AS1 knockdown alters the genomic distribution and abundance of H3K36me3

**Figure 3.**
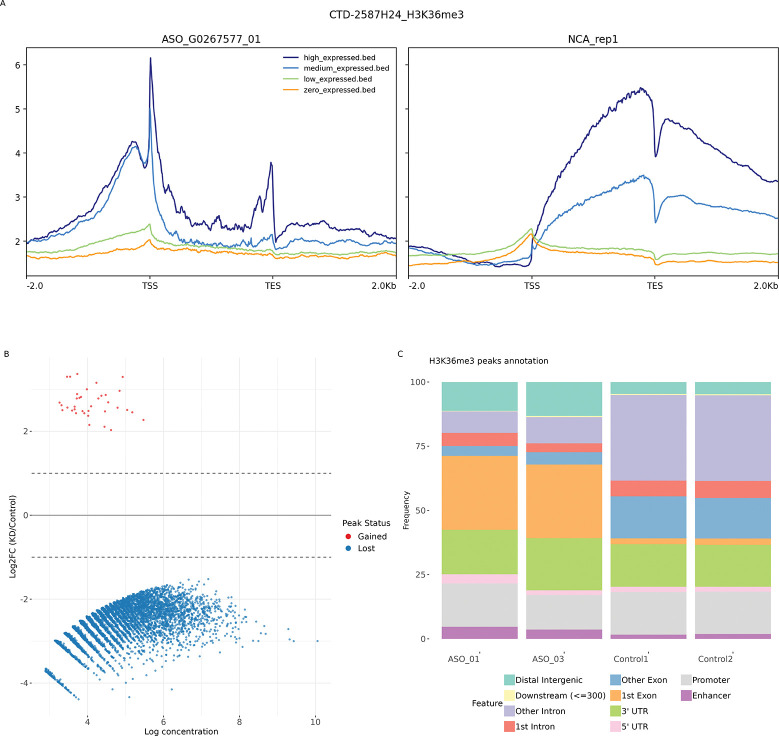
H3K36me3 ChIP-seq results for DNAAF3-AS1 knockdown and control **A** Metagene profile for DNAAF3-AS1 knockdown using ASO 01 and control **B** Differential binding analysis results, comparison knockdown and control, FDR < 0.05, |*log*2*FC*| > 1 **C** Genome annotation for DNAAF3-AS1 knockdown and control H3K36me3 peaks.

To experimentally validate the role of DNAAF3-AS1 in the regulation of the distribution of H3K36me3, we performed the H3K36me3 ChIP-seq in fibroblast cells following knockdown of DNAAF3-AS1 using two different antisense oligonucleotides (ASO 01 and ASO 03), with a non-targeting ASO as a control. Metagene analysis of highly expressed fibroblast genes in control revealed the canonical H3K36me3 distribution pattern, characterised by a sharp increase in signal intensity at the TSS, enrichment throughout the gene body, and a rapid decrease after the transcription end site (TES) (Figure 3A). In contrast, the metagene profile from DNAAF3-AS1 knockdown samples deviated from this pattern; the H3K36me3 signal distribution was indistinguishable from the input background.

Differential peak analysis further confirmed a significant loss of H3K36me3 peaks after knockdown (FDR < 0.05, |*log*2*FC*| > 1, Figure 3B). The genomic annotation of the peaks showed that in control cells, approximately 20% of the peaks were localised to promoter regions (−2000 to +2000 bp relative to TSS), with the majority residing in gene bodies (Figure 3C). Following DNAAF3-AS1 knockdown, we observed a notable redistribution: the proportion of peaks within the first exon increased, while the proportion in downstream exons and introns decreased. Concurrently, the proportion of peaks in intergenic regions increased. These findings align with the correlation data from the HiMoRNA database, confirming that depletion of DNAAF3-AS1 leads to an increased proportion of H3K36me3 peaks in intergenic regions and the first exon and a reduction of H3K36me3 in the remaining part of the gene body.

### The loss of DNAAF3-AS1 can lead to promoter switching

**Figure 4.**
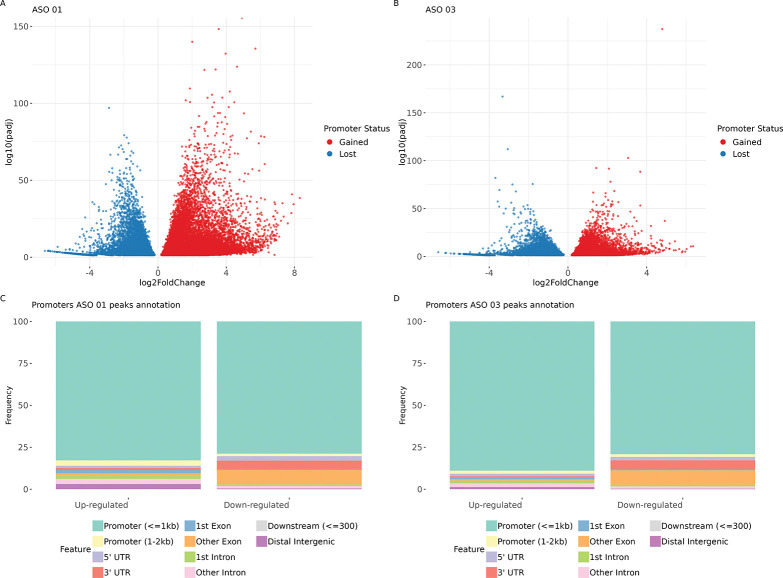
FANTOM6 differential expression analysis. **A,B** Volcano plots for differential expression analysis results for ASO 01 (**A**) and ASO 03 (**B**) **C,D** Genome annotation for differentially expressed promoters for ASO 01 (**C**) and ASO 03 (**D**).

Differential expression analysis for promoter regions from FANTOM6 was obtained for ASO 01 and ASO 03 compared to controls (FDR < 0.05, Figure 4A,B). The majority of upregulated promoters for both ASO replicates were located in the promoter region < 1 kb from the TSS (Figure 4C,D). However, for downregulated promoters for both replicas, an increased proportion was observed in the 3’UTR region and in exons other than the first. Thus, knockdown of DNAAF3-AS1 may cause disabling of promoters downstream of the TSS. Such promoter switching may occur due to alterations in the splicing process connected to the disrupted distribution of H3K36me3 marks.

## Discussion

The correlation analysis of HiMoRNA, the bias in the genomic annotation of the promoter after DNAAF3-AS1 knockdown, and the validation of the distribution of H3K36me3 after DNAAF3-AS1 knockdown indicate a regulatory role for this lncRNA in the establishment of the histone H3K36me3. This study did not determine whether this RNA regulates the mark-writing process or regulates erasers. The H3K36me3 histone mark is established by the histone methyltransferase SET domain containing 2 (SETD2). SETD2 is recruited into the transcription machinery during transcription elongation through an interaction of the SETD2 domain with the phosphorylated C-terminal domain (CTD) of RNA polymerase II, and thus can be propagated along gene bodies [34]. Additionally, SETD2 can interact with transcription elongation factors SPT6 and IWS1 [35], where SPT6 mediates an interaction with SETD2 bound to a nucleosome [20]. The slowing of RNA polymerase II by co-transcriptional splicing allows more H3K36me3 to be deposited at exons. This enrichment occurs preferentially at exons and towards the 3’ ends of genes [29].

The processes of establishing H3K36me1 and H3K36me2 are mainly regulated by the NSD1, NSD2, NSD3 and ASH1L proteins independently of SETD2 activity. However, there is evidence that loss of function of NSD proteins may influence H3K36me3 levels [13]. H3K36me3 was also identified in atypical heterochromatin simultaneously enriched with H3K9me3, while with removal of SETDB1, the H3K9me3 writer protein, such domains lose both marks and gain signatures of active enhancers.

The H3K36 methylation is enzymatically removed from chromatin by members of histone demethylase protein families containing the Jumonji domain, such as KDM4A, KDM4B and KDM4C [28]. The H3K36me3 mark can also be removed by other mechanisms, including histone turnover performed by ATP-dependent chromatin remodelling complexes and histone chaperones.

Redistribution of H3K36me3 may be directly caused by the interaction of DNAAF3-AS1 with one of the readers or writers of the histone mark or may be mediated by the regulatory function of DNAAF3-AS1 and the change in expression of one of the H3K36me3 regulators. One of the potential targets for DNAAF3-AS1 is HOTAIR, which is significantly upregulated in knockdown in both ASO experiments in FANTOM6. HOTAIR has been associated with inhibiting SETD2 transcription and reducing H3K36me3 levels [17]. Our study demonstrates that DNAAF3-AS1 is a key regulator of the H3K36me3 histone mark, directing its proper distribution across the genome. Knockdown of DNAAF3-AS1 leads to a redistribution of H3K36me3 and alters promoter usage. DNAAF3-AS1 function may be mediated through interactions with core regulators like SETD2 or other regulators such as HOTAIR, positioning it as a novel player in chromatin-based gene regulation.

## Supplementary Material

Supplement 1
