# Supplementary material for: Functional characterization of DNAAF3-AS1 in chromatin remodeling and H3K36me3 distribution
Source: bioRxiv. 2025 Dec 16:2025.12.15.694275. Preprint. [Version 1] doi: 10.64898/2025.12.15.694275 (PMC12723743; doi:10.64898/2025.12.15.694275)
Supplement: Supplement 1 [file media-1.pdf]

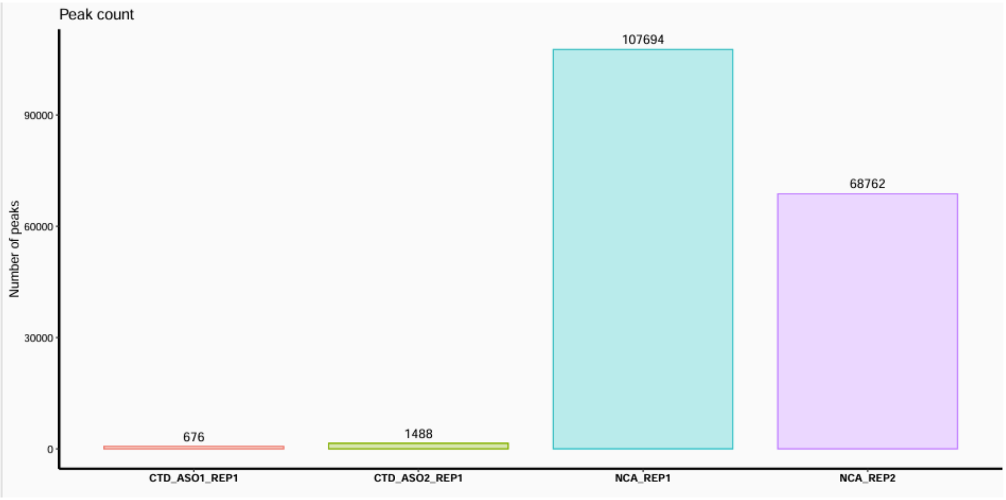

Supplementary Figure 1. Number of peaks obtained for each ChIP-seq sample with MACS3, q-value < 0.1.

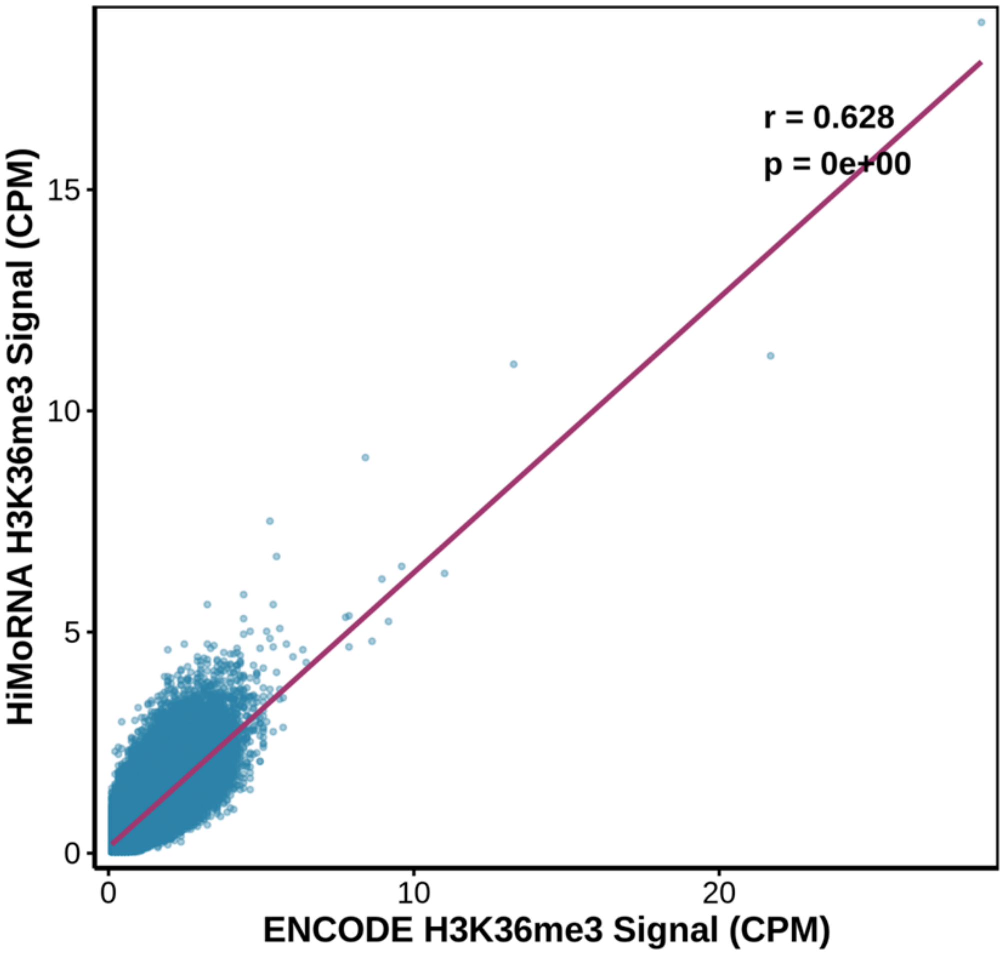

Supplementary Figure 2. Correlation of ChIP-seq samples signal and ENCODE H3K36me3 signal.
